# The Relationship Between Suicide Risk and Ruminative Thought in Alcohol and Substance Intoxication Cases Presenting to the Emergency Department

**DOI:** 10.3390/jcm15124805

**Published:** 2026-06-21

**Authors:** Serdar Derya, Ahmet Kutur, Mustafa Safa Pepele, Funda Kavak Budak

**Affiliations:** 1 Emergency Medicine Department, Inonu University, Malatya 44069, Turkeysafa.pepele@inonu.edu.tr (M.S.P.); 2 Emergency Medicine Department, Elazığ Training and Research Hospital, Elazığ 23000, Turkey; 3 Psychiatric Nursing Department, Inonu University, Malatya 44000, Turkey

**Keywords:** emergency department, rumination, suicide probability, substance use, alcohol intoxication

## Abstract

**Objective:** This study was conducted to examine the relationship between ruminative thinking styles and suicide probability in individuals presenting to the emergency department with suspected alcohol and substance intoxication/use, and to investigate whether these variables differ according to various demographic characteristics. **Methods:** The sample of this descriptive study consisted of 45 cases presenting to the emergency departments of two hospitals in eastern Turkey. Data were collected using a Sociodemographic Data Form, the Rumination Scale, and the Suicide Probability Scale. Descriptive statistics, Pearson correlation, independent samples *t*-test, and Linear Regression analysis were used for data analysis. **Results:** Of the participants, 66.7% were male and 44.4% were in the 18–23 age group. A positive and moderately significant relationship was found between rumination and suicide probability (r = 0.441; *p* = 0.001). Regression analysis revealed that rumination explained 34% of the variance in the suicide probability. Furthermore, suicide probability scores of those using non-alcohol or multiple substances were significantly higher than those using only alcohol (*p* = 0.025). **Conclusions:** Ruminative thinking is a significant associated factor of suicide risk in patients with alcohol and substance use disorders presenting to the emergency department. It is recommended that cognitive assessments of these patients be conducted during clinical processes and that multiple-substance users, in particular, should be closely monitored for suicide risk.

## 1. Introduction

Alcohol and substance use disorders are major social problems affecting both individuals and society. In addition to its psychological and physical impacts, alcohol and substance use is a biopsychosocial issue that affects individuals across multidimensional domains, including crime, accidents, homicide, suicide, unemployment, economic losses, social impairment, and family dissolution [1]. According to the UNODC 2020 report, 35.6 million people have used illicit substances at least once in their lives [2]. According to the World Health Organization (WHO), approximately 450,000 people lost their lives due to alcohol and substance use in 2015, and 167,750 of these deaths were directly related to drug use, primarily due to overdoses [3]. This situation leads to a high frequency of alcohol or substance intoxication cases, particularly in emergency department presentations. In addition to physical problems, psychiatric issues (depression, anxiety, psychotic disorders, etc.) are frequently observed in cases of alcohol and substance intoxication presenting to the emergency department [4]. One of the most serious psychiatric consequences of alcohol and substance use is suicide.

Approximately 160 million people worldwide have experienced suicidal ideation at least once in their lives, and more than 800,000 people die by suicide each year [5]. While all substances increase the risk of suicidal behavior, alcohol (22%) and opioids (20%) are the most common substances detected in individuals who die by suicide [6]. Over the past 20 years, increases have been observed in both alcohol consumption and suicide rates [7]. Alcohol and substance use weaken judgment and impulse control, which in turn increases the risk of suicidal behavior [8]. Therefore, a high risk of suicide can be observed in individuals presenting to the emergency department with alcohol or substance intoxication. Recent research indicates that suicidal behavior does not solely comprise impulsive acts but is also closely associated with ruminative thinking styles [9,10]. 

Ruminative thought is understood as a style of thinking about an individual’s problems or negative experiences, characterized by five core features: the thinking is repetitive, at least partially intrusive, difficult to disengage from, perceived as unproductive, and captures mental capacity [11]. Rumination arises in the context of various emotional disorders, such as depression, eating disorders, and alcohol use disorders [12]. Studies have demonstrated the relationship between ruminative thinking and suicide [10,11,13]. The cognitive process in rumination may function as a mechanism that supports the persistence of suicidal ideation, particularly in individuals experiencing stressful life events or using drugs [11,13].

When alcohol and substance use are combined with rumination, an individual’s capacity to cope with negative emotions may decrease. This situation increases the difficulties in emotional regulation and creates a cognitive trap in the process, leading to suicidal behavior. Understanding these cognitive patterns in cases of alcohol and substance intoxication observed in emergency departments is critical for both clinical observation and intervention.

The primary objective of this study was to examine the relationship between the level of ruminative thought and suicide risk in individuals presenting to the emergency department due to alcohol or substance intoxication. Revealing this relationship contributes to understanding the cognitive processes underlying suicidal behavior and ensures that risk assessment in the emergency department is conducted more holistically.

### Research Questions

1. What is the mean total score of the Suicide Probability Scale among alcohol and substance intoxication cases presenting to the emergency department?
2. What is the mean total score of the Ruminative Thinking Scale among patients with alcohol and substance intoxication presenting to the emergency department?
3. Is there a relationship between the mean total scores of the Suicide Probability Scale and the Ruminative Thinking Scale among individuals experiencing alcohol and substance intoxication presenting to the emergency department?

## 2. Materials and Methods

Study Design: This retrospective, descriptive, and correlational study was conducted in a single center.

Setting and Period: The study was conducted between September 2025 and November 2025 with patients presenting to the emergency departments of two hospitals in eastern Turkey.

Population and Sample of the Study: The study population consisted of patients who presented with alcohol or substance intoxication to the emergency departments of two hospitals in eastern Turkey and were discharged for at least one month. Patient information was retrospectively accessed through the hospital records system.

The sample size was calculated using a 5% margin of error and 90% statistical power, assuming a moderate-to-large effect size (Cohen’s d = 0.65) based on previous studies examining rumination and suicide risk [14]. This calculation indicated that 36 patients were required. To account for potential data loss, 40 individuals were included. The study included 45 patients.

Inclusion criteria were as follows: age > 18 years, at least one month since the alcohol or substance intoxication event, and accessible medical records with sufficient data.

Exclusion criteria were as follows: individuals with severe cognitive impairment (dementia, severe neurological disease, etc.).

Withdrawal criteria were as follows: cases in which the participant’s medical records, survey responses, or scales were missing, incorrect, or insufficient for analysis.

### 2.1. Data Collection Tools

Sociodemographic Characteristics Form: This form was used to query the sociodemographic characteristics of the individuals, including age, sex, marital status, educational level, income level, employment status, and type of substance used.

Suicide Probability Scale (SPS): Originally developed by Cull and Gill (1990), the Turkish reliability and validity study of the scale in patients with depression was conducted by Batıgün and Şahin (2003) [15,16]. The scale consists of 36 items and is scored on a 4-point Likert-type scale. The participants were asked to evaluate the items based on percentages. Items 2, 6, 7, 10, 11, 18, 20, 21, 22, 24, 25, 26, 27, 30, 32, 35, and 36 are reverse-coded. Although separate total scores can be obtained for each subscale, the sum of all scores provides the general suicide probability score. The total score obtainable from the scale ranges from 36 to 144, with higher scores indicating a higher probability of suicide. In the Turkish validity and reliability study, Cronbach’s alpha coefficient was determined to be 0.86. In the present study, Cronbach’s alpha coefficient was 0.87.

Ruminative Thought Style Questionnaire (RTSQ): Developed by Brinker and Dozois (2009), the Ruminative Thought Style Questionnaire is an assessment tool aimed at measuring general ruminative thought patterns that are not limited to specific psychopathological conditions, treating rumination as a cognitive process independent of psychopathology [17]. The scale consists of 20 items rated on a 7-point Likert-type scale (1 = does not describe me at all; 7 = describes me perfectly), providing a total score range of 20–140, with higher scores indicating greater ruminative tendencies. The Turkish adaptation, validity, and reliability study was conducted by Karatepe (2010), and the Cronbach’s alpha internal consistency coefficient for the overall Turkish form was 0.91 [17,18]. In the present study, Cronbach’s alpha coefficient was 0.94.

### 2.2. Data Collection

The researcher collected data between September 2025 and December 2025 at the emergency clinics of two hospitals in eastern Turkey. Patients who presented to the emergency clinic with alcohol or substance intoxication and whose discharge occurred at least one month prior were identified through the hospital record system. Patients were contacted via telephone. The data collection tools were administered by inviting the patients to the hospital for a follow-up visit or by conducting home visits. The administration of the data collection tools took approximately 20 min.

### 2.3. Statistical Analysis of Data

The SPSS 20 statistical software package was used to analyze the data. The participants’ descriptive characteristics were presented using frequencies and percentages. Descriptive statistics for the scores obtained by the individuals on the scales are presented as means and standard deviations. The normality of the scale scores was checked using the Shapiro–Wilk test, and kurtosis and skewness values were also examined. For the analysis of data determined to follow a normal distribution, one-way ANOVA, independent samples *t*-test, Pearson correlation analysis, and multiple regression analysis were used. The study data were analyzed at a 95% confidence level, and a *p*-value < 0.05 was considered statistically significant.

### 2.4. Ethical Considerations

Prior to the commencement of the study, approval was obtained from the Scientific Ethics Committee of Health Sciences (Approval No.: 2025/8783) and legal permission was granted by the hospitals where the research was conducted.

## 3. Results

Of the patients participating in the study, 66.7% were male, 44.4% were in the 18–23 age group, 42.2% were married, and 40% reported using alcohol and experiencing alcohol-related intoxication. Additionally, 57.7% were secondary school graduates, 88.8% were employed, and 100% had middle-level income (Table 1).

A significant difference was found between the type of substance used and the suicide probability. The suicide probability scores of those using multiple substances or non-alcoholic substances were significantly higher than those using only alcohol (*p* < 0.01; Table 2).

Regression analysis was conducted to examine the associated factors of suicide probability among participants. Age, gender, substance use type, and rumination were initially entered into a stepwise regression model. In the final model, rumination was retained as a significant associated factor, whereas age, gender and substance use were not significant. The model explained 34% of the variance in suicide probability (R^2^ = 0.34; Adjusted R^2^ = 0.32; *p* = 0.001). Higher rumination scores were associated with increased suicide probability. Using a stepwise approach allowed for a more accurate estimation of the unique contribution of rumination while controlling for other relevant variables (Table 3).

The analyses revealed that ruminative thinking styles directly predicted a 19.5% increase in suicide probability in individuals presenting to the emergency department with suspected intoxication. A positive, moderate, and statistically significant relationship was found between ruminative thoughts and suicide probability (r = 0.441; *p* = 0.001). In other words, as the tendency for ruminative thoughts increases, the probability of suicide also increases, or as the probability of suicide increases, the tendency for ruminative thoughts also increases (Table 3).

## 4. Discussion

This study examined the relationship between ruminative thinking styles and suicide probability in individuals presenting to the emergency department with suspected alcohol and substance intoxication. The findings support the research questions.

When evaluating the mean total score of the Rumination Scale in the study, it can be stated that the patients’ scores were above average or high. This elevated trend suggests that individuals experiencing substance use or intoxication are cognitively “stuck” in the crisis they face. In a study conducted by Weterberg et al. (2026) with individuals with alcohol and substance use disorders, rumination was directly associated with substance craving and the inability to manage negative affect [19]. The high average observed in the present study demonstrates that these individuals cannot escape the cycle of repetitive thinking, even while under the influence of a substance or immediately afterward, which creates a cognitive load that may exacerbate the medical condition.

Furthermore, when examining the mean total score of the Suicide Probability Scale, it can be observed that the patients’ scores were above average and high. This indicates that emergency department presentations are not merely cases of “accidents” or “overdoses” but harbor a serious underlying potential for suicide. According to the “Cry of Pain” model proposed by Williams et al. (2007), the probability of suicide increases when an individual feels an inescapable sense of pain and defeat [20]. Our mean score of 91.91 points provides quantitative evidence that this group of patients feels cognitively trapped and helpless.

The most fundamental finding of this study was the identification of a positive and statistically significant relationship between ruminative thoughts and suicide probability. The regression analysis results showed that rumination alone explained approximately 34% of the variance in suicide risk. This situation reveals that an individual’s entry into a continuous, repetitive thought cycle by becoming stuck on past negative experiences or current problems is a critical cognitive process that triggers or reinforces suicidal ideation. Watkins and Roberts (2020) stated that rumination exhausts an individual’s cognitive resources and dulls “flexible thinking” and “effective problem-solving” skills [13]. This cognitive blockage may cause the individual to be unable to find a way out of the psychological pain they are experiencing and ultimately perceive suicide as the only “solution”. According to the “Cry of Pain” model proposed by Williams et al. (2007), rumination is a catalytic process that leads the individual to feel “trapped” [20]. The 34% predictive power revealed in this study statistically supports the notion that the repetitive thought cycle transforms this sense of entrapment into a concrete suicide risk by nourishing it. As highlighted in the systematic review conducted by Nock and O’Connor (2014), rumination acts as a “moderator” that strengthens the relationship between hopelessness and suicidal ideation [21].

Another noteworthy finding of this study is the difference in suicide risk between those who use alcohol only and those who use other substances or multiple substances. The analyses demonstrated that the suicide probability scores of cases presenting to the emergency department due to multiple-substance use or non-alcohol substance use were significantly higher than those experiencing only alcohol intoxication. The significance of this relationship, particularly in cases presenting to the emergency department due to alcohol and substance use, is critical. When the impulsivity brought on by the substance is combined with the cognitive fixation caused by rumination, the likelihood of an individual engaging in self-harm increases exponentially [10]. This situation strengthens our inference that an individual’s fixation on past negative experiences is not merely a psychological disturbance but a "critical cognitive process" that is life-threatening during moments of acute crisis.

Another important conceptual perspective that should be considered when interpreting these findings is the possibility that both substance use and suicidal behavior may function as maladaptive coping responses to intrusive ruminative thoughts. Individuals experiencing persistent and repetitive negative thinking may attempt to regulate overwhelming emotional distress through substance use as a form of short-term cognitive or emotional escape [21,22]. Similarly, suicidal ideation or behavior may emerge as an extreme form of problem-solving when individuals perceive their distress as uncontrollable and inescapable [21]. From this perspective, substance use and suicidality may represent not only risk behaviors but also attempts to manage the cognitive and emotional burden created by rumination. This interpretation is consistent with theoretical models suggesting that maladaptive coping strategies arise when individuals lack sufficient cognitive flexibility and emotional regulation skills [22]. Therefore, the elevated rumination levels observed in this study may contribute to both increased substance use severity and heightened suicide probability by driving individuals toward ineffective coping strategies during acute crises [21,22].

## 5. Conclusions and Recommendations

This study revealed the critical role of rumination, a cognitive process, in predicting suicide risk among individuals presenting to the emergency department due to alcohol and substance intoxication/use. The results indicate that mental repetition of negative experiences (rumination) significantly increases the probability of suicide. This suggests that suicide risk is not merely an instantaneous crisis but also the result of a chronic cycle of negative thoughts. Furthermore, individuals presenting to the emergency department for multiple or illicit substance use had significantly higher suicide probability scores compared to those using alcohol alone. This finding suggests that substance diversity may be associated with a greater clinical vulnerability, potentially complicating the individual’s ability to utilize adaptive coping mechanisms during crises. Rather than a direct causal erosion of resilience, the use of multiple substances may reflect an underlying severity of psychological distress that manifests as both higher ruminative tendencies and increased suicide risk.

Short-term psychoeducation, incorporating evidence-based techniques, such as cognitive restructuring for ‘coping with cognitive distortions’ and ‘rumination-focused cognitive behavioral therapy techniques’, is recommended for patients identified with high suicide risk before discharge [13]. Furthermore, identifying specific demographics—particularly single young adults with poly-substance use—as a ‘high-priority’ group aligns with clinical guidelines suggesting that multi-modal risk factors require immediate psychiatric consultation (APA Practice Guidelines). The integration of ruminative thought as an associated factor, which explained 34% of the variance in this study, provides a measurable clinical metric for such early warning systems.

### Limitations of the Study

The study was limited to 45 patients who presented to the emergency department. The relatively small sample size limits the generalizability of the findings to the entire population of alcohol and substance users. Studies with larger sample sizes will increase the statistical power and allow for a clearer observation of the relationships between variables. A limitation of the study is the relatively small sample size, which may limit the generalizability of the findings. Future studies with larger samples are recommended. Another limitation is that the data were collected using scales based on the participants’ self-reports.

## Figures and Tables

**Table 1:** Descriptive characteristics of patients.

Characteristics	*n*	%
Gender		
Male Female	30 15	66.7 33.3
Age Group		
18–23 24 and above	20 25	44.4 55.6
Marital Status		
Married Single/divorced	19 26	42.2 57.8
Educational Status		
Secondary school High school and above	26 19	57.7 42.3
Employment Status		
Employed Unemployed	40 5	88.8 11.2
Income Level		
Middle	45	100.0
Type of Substance/Intoxication		
Alcohol Non-alcohol/multiple substances	18 27	40.0 60.0
Total	45	100.0

**Table 2:** Comparison of scale scores according to descriptive characteristics.

Variable		Rumination Total (Mean ± SD) (114.62 ± 14.49)	Statistical Significance and *t*	Suicide Probability (Mean ± SD) (92.20 ± 7.85)	Statistical Significance and *t*
Gender	Male Female	114.1 ± 14.2 115.3 ± 15.0	*t*: −0.27 *p*: 0.790	92.4 ± 7.9 91.9 ± 7.7	*t*: 0.20 *p*: 0.843
Marital Status	Married Single	113.8 ± 12.8 115.2 ± 15.7	*t*: −0.32 *p*: 0.748	91.5 ± 7.5 92.7 ± 8.1	*t*: −0.51 *p*: 0.611
Substance Use Type	Alcohol Other/Multiple	112.4 ± 13.1 118.9 ± 16.2	*t*: −1.41 *p*: 0.167	90.8 ± 7.2 94.9 ± 8.3	*t*: −1.72 ***p*: 0.024**

*p* < 0.05 level of significance. Note: An independent samples *t*-test was used for two-group comparisons.

**Table 3:** Correlation and stepwise regression analysis findings of factors predicting suicide probability.

Associated Factor	β	SE	*t*	*p*	Correlation
Age	0.05	0.06	0.83	0.41	
Gender	0.12	0.07	1.71	0.83	
Substance use type	0.35	0.08	1.38	0.17	
Rumination	0.18	0.07	4.29	0.001	r: 0.441 *p*: 0.001
Suicide probability	0.30	0.06	3.12	0.001	

The stepwise regression method was used. R^2^ = 0.34; Adjusted R^2^ = 0.32; F(3, 41) = 7.18; *p* < 0.001. β: standardized regression coefficient; SE: standard error; *t*: *t*-test value; *p*: significance level. *p* < 0.01 level of significance; Pearson correlation analysis.

## Data Availability

The data presented in this study are available upon request from the corresponding author (due to the need to protect the confidentiality of patient information).
